# Efficacy and Safety Assessment of ThriveCo Scar Fader Gel With Scarcede™ in the Treatment of Skin Scars

**DOI:** 10.7759/cureus.90934

**Published:** 2025-08-25

**Authors:** Maheshvari N Patel, Nayan Patel, Apeksha Merja, Saurav Patnaik, Shatakshi Maulekhi

**Affiliations:** 1 Clinical Research, NovoBliss Research Private Limited, Ahmedabad, IND; 2 Pharmacology, Swaminarayan University, Ahmedabad, IND; 3 Clinical Research Operations, NovoBliss Research Private Limited, Ahmedabad, IND; 4 NovoBliss Research Private Limited, Clinical Trials, Ahmedabad, IND; 5 Cosmetology, Anveya Living Private Limited, Gurgaon, IND

**Keywords:** acne scar, copper tripeptide, hesperidine, skin scars, sodium hyaluronate

## Abstract

Introduction

Skin scars, a natural outcome of dermal injury healing, may seem minor but often lead to significant disfigurement and psychological distress. This study aimed to assess the safety and efficacy of ThriveCo Scar Fader Gel containing Scarcede™ (Anveya Living Private Limited, Bengaluru, India), a proprietary blend of copper tripeptide-1, L-carnitine L-tartrate, alpha-glucosyl hesperidin, and sodium hyaluronate. This synergistic formulation targets multiple scar-improvement mechanisms, offering potential for enhanced skin texture and minimized scar appearance.

Method

An open-label, interventional, prospective real-world evidence study was conducted to evaluate the clinical safety, efficacy, and in-use tolerability of the test product in females with acne scars. Ethical approval was granted by the Independent Ethics Committee, and informed consent was obtained from all participants. Visible scar changes were assessed using the Manchester Scoring Scale by a dermatologist. Skin texture changes, including smoothness, roughness, and scaliness, were evaluated using VISIOSCAN® VC20 Plus (Courage + Khazaka electronic GmbH, Cologne, Germany). Digital photographs documented progressive scar visibility reduction. Statistical analyses were performed using IBM SPSS Statistics for Windows, Version 29.0.1.0 (Released 2022; IBM Corp., Armonk, New York) and Microsoft® Excel 2019 (Microsoft Corporation, Redmond, Washington), with significance reported at the 5% level.

Result

The test product reduced scar appearance by 7.17% at day 21 (p<0.001) and 26.57% at day 45 (p<0.0001). Skin smoothness improved by 33.08% at day 21 (p<0.0001) and 44.56% at day 45 (p<0.0001). Skin roughness decreased by 100.86% at day 21 (p<0.0001) and 161.62% at day 45 (p<0.0001). Scaliness reduced by 39.71% at day 21 (p<0.001) and 58.92% at day 45 (p<0.0001). No local or systemic adverse effects were reported.

Conclusion

ThriveCo Scar Fader Gel with Scarcede™ demonstrated clinical safety and efficacy in scar reduction, supporting smoother, clearer skin without adverse effects and suitable for routine skincare use.

## Introduction

Scar formation is a frequent outcome of skin injuries, often resulting in various negative effects such as physical deformities and psychological challenges [1]. The biomechanical properties of skin vary depending on direction, as they are shaped by the alignment of its collagen-rich fibers and the viscous gel-like matrix surrounding them. When the skin is injured, its structure and composition are altered, which significantly impacts the mechanics and alignment of the scar tissue that forms [2]. Each year, approximately 100 million patients in developed countries develop scars, many of which pose significant challenges. Individuals with abnormal scarring may face a range of complications, including social or psychological burdens [3]. Acne scarring often occurs due to delayed or insufficient medical intervention, although it can also develop despite proper treatment. The impact of acne scarring extends beyond cosmetic concerns, as it is frequently linked to mental health issues such as depression, suicidal thoughts, emotional distress, low self-esteem, embarrassment, and social difficulties, all of which can severely affect a person’s overall well-being [4].

Hyaluronic acid (HA) is a naturally occurring glycosaminoglycan that plays a key role in maintaining the skin's moisture balance. While it is widely known for its use in facial volumization and reducing wrinkles, recent advancements have focused on invasive HA formulations for enhancing skin quality and texture, offering broader applications in skin rejuvenation [5]. Hyaluronan serves a variety of functions, primarily due to its hygroscopic properties, which significantly impact hydration and the physical characteristics of the extracellular matrix (ECM) [6]. Hesperidin, a prominent natural flavonoid, is recognized for its antioxidant, anti-inflammatory, anti-mutagenic, and anti-hypertensive properties, yet it remains underexplored in skincare products, with limited literature available on its stability within topical formulations [7]. Furthermore, it improves epidermal permeability barrier function and maintains homeostasis in both youthful and aging skin. The beneficial effects of hesperidin on cutaneous functions are primarily attributed to its potent antioxidant activity. Due to its low cost, widespread availability, and excellent safety profile, hesperidin holds significant potential as a therapeutic agent for managing various dermatological conditions [8].

In addition, L-carnitine offers potential benefits for the skin, including enhancing skin hydration, reducing signs of aging, improving skin elasticity, and promoting overall skin health by supporting cellular energy production and fat metabolism within skin cells [9]. Furthermore, peptides are widely valued in enhancing skin conditions, offering antioxidant, anti-inflammatory, collagen-synthesizing, anti-wrinkle, and wound-healing benefits. Recent research highlights their role in supporting the dermal-epidermal junction (DEJ), a key interface that provides structural integrity, facilitates molecular transport, and influences skin aging by targeting basement membrane signals [10]. Copper tripeptide-1 is essential for accelerating wound healing by initiating repair processes during tissue injury. It has proven effective in various wounds, including surgical, post-laser, burns, and transplants, with healing occurring three times faster in its presence. Additionally, it supports primary healing and tissue remodeling by stimulating keratinocyte proliferation, collagen synthesis, and improving skin thickness, elasticity, and firmness, while reducing wrinkles, photodamage, and pigmentation, and enhancing skin clarity and barrier function [11].

Together, copper tripeptide-1, L-carnitine L-tartrate, alpha-glucosyl hesperidin, and sodium hyaluronate play a crucial role in providing potent antioxidant, anti-inflammatory, and skin-healing benefits. These ingredients work synergistically to improve skin health, reduce inflammation, and enhance the overall appearance and texture of the skin. However, advancements in skincare, including the use of HA, hesperidin, and L-carnitine, offer promising solutions for improving skin quality, texture, and overall health. These treatments, with their multiple benefits, represent valuable tools in addressing various skin conditions and enhancing skin regeneration.

This study aimed to evaluate the safety, efficacy, and in-use tolerability of Scar Fader Gel in healthy adult female subjects with acne scars. The primary objective was to assess the effectiveness of the test product in reducing visible scars, as measured by changes from baseline to post-treatment. Secondary objectives included evaluating improvements in skin texture parameters, such as smoothness, roughness, and scaliness, from baseline to the end of the treatment period. Digital photographs were also analyzed to document changes in scar appearance before and after the use of the test product.

## Materials and methods

Ethical conduct of study

The clinical investigation, including the informed consent document (ICD), was reviewed and approved by the ACEAS Independent Ethics Committee. The ACEAS Committee is registered with the Central Drugs Standard Control Organization (CDSCO) (registration# ECR/281/Indt/GJ/2017/RR-21) and the OHRP US DHHS (registration# IRB00011046). The study protocol (Ver#1.0), along with the English and Gujarati versions of the ICD (Ver#1.0), case report form (CRF) (Ver#2.0), and other pertinent documents, received approval from the ACEAS Independent Ethics Committee on December 5, 2023, prior to the initiation of the study. The study was conducted in strict compliance with established standard operating procedures (SOPs), the approved protocol, any protocol amendments, and applicable Good Clinical Practice (GCP) standards for clinical research in India (2005), the New Drugs and Clinical Trials Rules 2019, ICH E6 (R2) Guidelines on Good Clinical Practice, the ICMR National Ethical Guidelines for Biomedical and Health Research Involving Human Participants (2017), and the Declaration of Helsinki (Brazil, October 2013). This clinical trial has been registered with the Clinical Trial Registry of India (CTRI) under the registration number CTRI/2024/01/061138, registered on January 5, 2024.

Study design

This study was an open-label, interventional, prospective clinical study designed to evaluate the safety and efficacy of Scar Fader Gel. The study was conducted at Contract Research Organization - NovoBliss Research Private Limited, Ahmedabad, India, involving a total of 32 healthy adult females aged 18 to 60 years with skin scars. The clinical study treatment duration was 45 days.

The product’s effectiveness was assessed by evaluating skin texture, with a specific focus on smoothness, roughness, and scaliness. Potential participants were females aged 18-60 years, healthy, non-pregnant, and non-lactating. They needed to have acne scars and be in good health based on recent medical history. The pre-screening process was carried out by the screening department at NovoBliss Research. Prior to the enrolment visit, participants were contacted by the recruiting team via telephone. During screening, participants were instructed not to use any skincare or scar reduction products on the application site on the study visit day, following a structured visit schedule as outlined in the protocol.

The participants were scheduled to visit the facility for three key appointments: visit 01 (Day 01) for screening and enrolment, visit 02 (Day 21 ± 2 days) for the test product usage period and evaluation, and visit 03 (Day 45 ± 2 days) for the end-of-study evaluation. Efficacy assessments included changes in scar appearance using the Manchester Scar Scale (MSS) scoring, skin texture changes (smoothness, roughness, scaliness) evaluated with Visioscan VC20Plus (Courage + Khazaka electronic GmbH, Cologne, Germany), and visual changes analyzed through digital photographs captured with a Nikon digital camera. Additionally, the Subject Response Index was measured on Day 1 and after using the test product on Day 21 (±2 days) and Day 45 (±2 days).

This study included healthy, adult, non-pregnant, non-lactating females aged 18 to 60 years with acne scars. Female participants of childbearing potential were required to have a negative urine pregnancy test and use reliable contraception for at least six weeks prior to and during the study. Exclusion criteria included active infections or dermatological conditions at the application site, allergies to the study treatment, recent use of systemic therapies, pregnancy or breastfeeding, prior scar treatments, substance abuse history, or participation in similar studies within the last four weeks.

Test product

The treatment used in the study was Scar Fader Gel. Participants were instructed to wash and pat dry the scar area before applying a thin layer of the gel to the affected area. They then gently massaged the product into the skin until it was fully absorbed. The gel contained a combination of copper tripeptide-1, L-carnitine L-tartrate, alpha-glucosyl hesperidin, and sodium hyaluronate. The treatment was applied twice a day via a topical route. The gel was manufactured by Anveya Living Private Limited.

Statistical analysis

Continuous variables were analyzed using descriptive statistics, including the number of observations (N), mean, standard deviation (SD), median, as well as the minimum and maximum values. Categorical variables were summarized by frequency and percentage, with graphical representations included where appropriate. Adverse events (AEs) were summarized in terms of both number and percentage. Statistical analyses were conducted using IBM SPSS Statistics for Windows, Version 29.0.1.0 (Released 2022; IBM Corp., Armonk, New York) and Excel software (Microsoft Corporation, Redmond, Washington), with a significance level set at 5%. Subjects who withdrew from the study were not included in the statistical analysis.

Sample size determination

The sample size for this study was determined based on insights from a thorough literature review, providing guidance on typical enrolment numbers for similar research. This approach ensured a balance between feasibility and scientific rigor, with a target of 30 completed subjects to account for potential dropouts. A total of 32 participants were enrolled, and 30 successfully completed the study, ensuring data robustness and reliability. The sample size was carefully selected to generate meaningful outcomes while maintaining a 95% confidence interval, reinforcing the validity and reliability of the study’s findings.

## Results

Demographic and baseline characteristics

**Figure 1:**
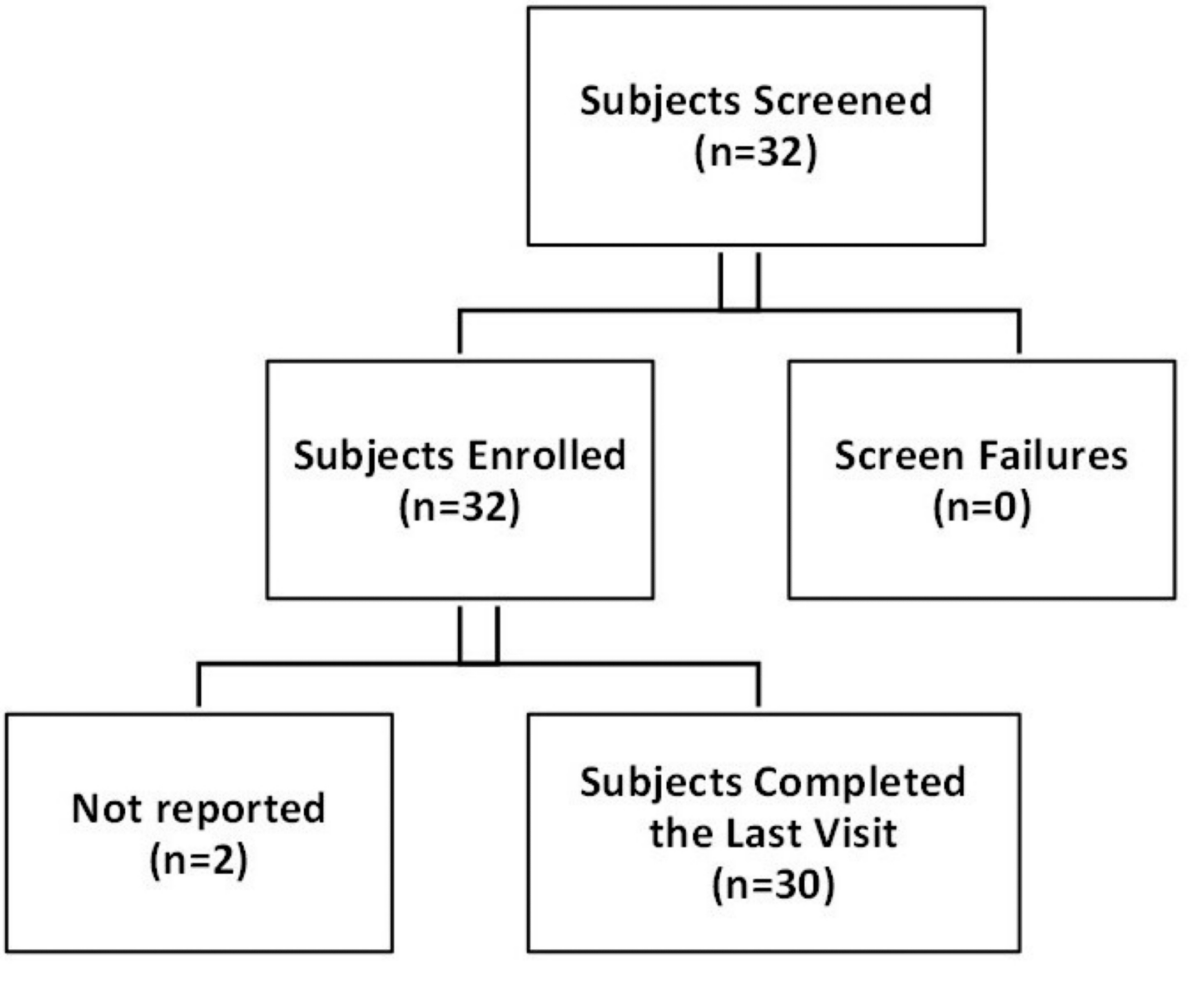
Subject Disposition

In this study, 32 female participants were enrolled; all 32 (100.00%) subjects received the test product. Out of the 32 subjects, 30 subjects had completed the study (Figure 1).

Table 1 presents the demographics of the subjects.

**Table 1:** Subject's Demographics

Parameters	Statistics	Enrolled subjects, N=32	Study completed subjects, N=30
Gender	Female	32 (100.00%)	30 (100.00%)
Predominant race	Asian	32 (100.00%)	30 (100.00%)
Medical/ConMed history	No	32 (100.00%)	30 (100.00%)
Age	N	32	30
	Mean	33.19	33.53
	SD	7.85	7.99
	Median	33.00	33.00
	Min	22.00	22.00
	Max	48.00	48.00
Height (cm)	N	32	30
	Mean	156.22	156.30
	SD	8.58	8.67
	Median	154.50	154.50
	Min	143.00	143.00
	Max	178.00	178.00
Weight (kg)	N	32	30
	Mean	60.25	60.47
	SD	11.46	11.81
	Median	58.50	59.50
	Min	40.00	40.00
	Max	98.00	98.00

Evaluation

The efficacy of the test product was assessed using several parameters. The Manchester Scar Scale (MSS) scoring was used to evaluate visible changes in scars, as recorded in the CRF. Digital photographs were taken to document changes in the affected areas. Additionally, the Visioscan® VC20Plus was used to assess changes in skin texture, including smoothness, roughness, and scaliness, as recorded in the CRF. Furthermore, the Subject Response Index was utilized to gather consumer perception data regarding the test product, capturing subjects’ responses and feedback about its effectiveness.

Primary efficacy endpoint: change in scar appearance - MSS

**Figure 2:**
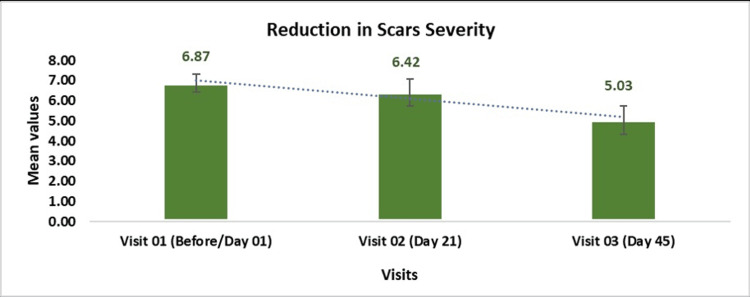
Mean Change in Visible Scars Associated by Manchester Scar Scale Evaluated by Dermatological Assessment The data are presented as mean ± SD. P-value is considered significant at <0.05.

Changes in visible scars, as assessed by the MSS, demonstrated a significant improvement over time, with a statistically significant reduction in mean scores. At baseline (Day 01), the mean score was 6.87 ± 0.43, which decreased to 6.42 ± 0.65 by Day 21 (p < 0.001, t = -4.15) and further declined to 5.03 ± 0.72 by Day 45 (p < 0.0001, t = -13.45). This represents a 1.07-fold reduction from baseline on Day 21 and a 1.36-fold reduction on Day 45. The calculated delta value of -1.83 underscores the substantial decrease in scar severity, highlighting the treatment’s effectiveness in achieving the targeted outcomes (Figure 2).

Secondary efficacy endpoints: assessment of smoothness, roughness, and scaliness by Visioscan® VC20Plus

Skin Smoothness

The change in smoothness, assessed by Visioscan VC20Plus, showed significant improvement from baseline, with a reduction in the mean score. On Day 01, the mean score was 286.43 ± 45.57, which decreased to 191.25 ± 35.41 on Day 21 (p < 0.0001, t = -13.20), and further improved to 156.74 ± 27.65 on Day 45 (p < 0.0001, t = -17.23). This represents a 1.50-fold improvement from baseline on Day 21 and a 1.83-fold improvement (up to 2×) on Day 45. These findings suggest that continuous use of the test product significantly improved skin smoothness.

Skin Roughness

The change in roughness, assessed by Visioscan VC20Plus, showed significant improvement from baseline, with an elevation in the mean score. At baseline on Day 01, the mean score was 1.29 ± 0.55, which increased to 2.11 ± 0.73 on Day 21 (p < 0.0001, t = 6.63), and further increased to 2.74 ± 0.93 on Day 45 (p < 0.0001, t = 8.34). This represents a 1.63-fold increase on Day 21 and a 2.12-fold increase on Day 45 from baseline, indicating that continuous use of the test product significantly reduced skin roughness.

Skin Scaliness

**Figure 3:**
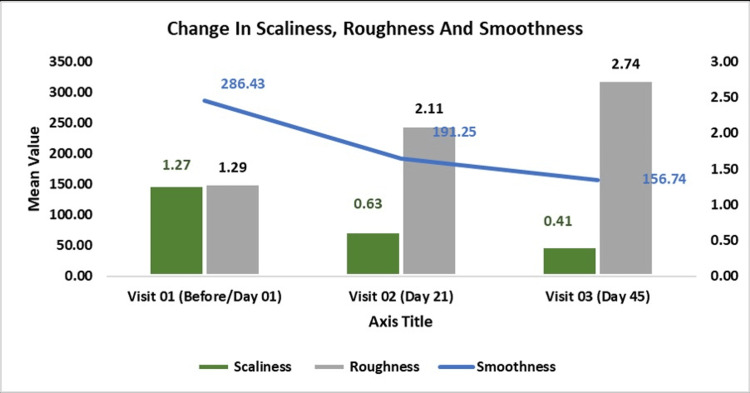
Mean Change in Scaliness, Roughness and Smoothness Assessed by Visioscan VC20 Plus. The data are presented as mean ± SD. P-value is considered significant at <0.05.

The change in scaliness, assessed by Visioscan VC20Plus, showed significant improvement from baseline, with a reduction in the mean score. On Day 01, the mean score was 1.27 ± 0.90, which decreased to 0.63 ± 0.37 on Day 21 (p < 0.001, t = -3.80), and further decreased to 0.41 ± 0.30 on Day 45 (p < 0.0001, t = -5.40). This represents a 2.02-fold reduction from baseline on Day 21 and a 3.08-fold reduction on Day 45. These findings suggest that continuous use of the test product significantly reduced skin scaliness (Figure 3).

Digital photographs

**Figure 4:**
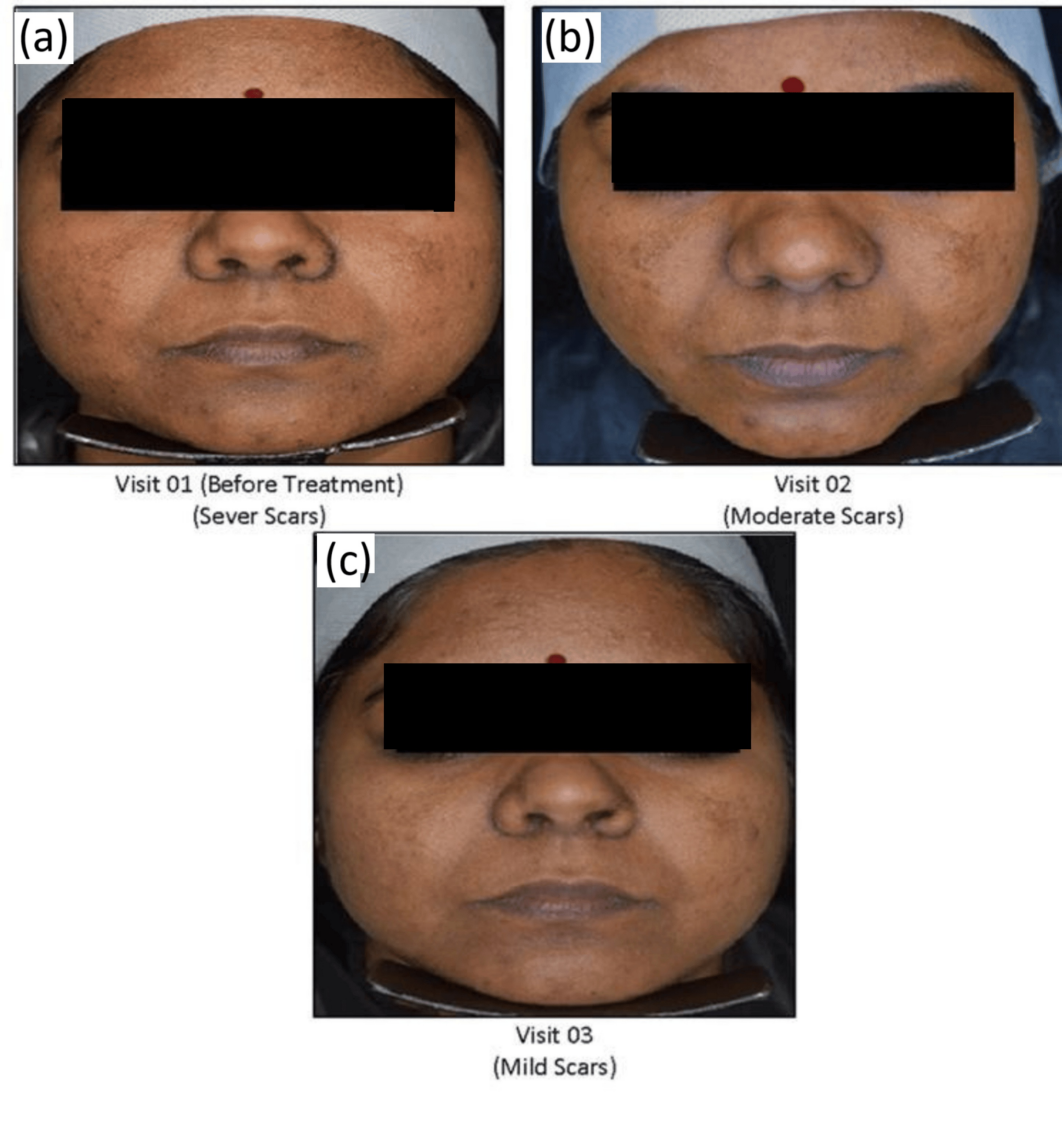
Digital Photographs (a) Before treatment use. (b) Moderate scars. (c) Mild scars.

Photographs of the application site for subject no. 010 were taken at baseline at Visit 1, when the scars were severe, and at Visit 2, after treatment, when the scars showed moderate visibility. By Visit 3, negligible scars were present. These images illustrate a progressive reduction in scar visibility throughout the treatment period (Figure 4).

Treatment perception questionnaire

Among the subjects, 2 (6.67%) had prior experience with scar treatments. When evaluating an earlier test product, 1 (3.33%) rated it moderately ineffective, and 1 (3.33%) considered it slightly effective in reducing scars. Similar mixed responses were noted for smoothness, dryness, roughness, redness, itchiness, and scaliness, with some participants rating the product as very ineffective or only slightly effective. Overall, 1 (3.33%) participant found the earlier product very ineffective, and another rated it slightly effective in terms of satisfaction. In contrast, the MSS evaluation in this study showed significant improvements over time, with scar appearance decreasing by 7.17% on Day 21 and 26.57% on Day 45. Skin smoothness improved by 33.08% on Day 21 and 44.56% on Day 45, while skin roughness and scaliness showed reductions of 100.86% and 161.62%, and 39.71% and 58.92% on Day 21 and Day 45, respectively. These findings indicate notable efficacy with the continued use of Scar Fader Gel.

## Discussion

Skin scarring encompasses a broad range of clinical phenotypes, from normal fine lines to more severe forms such as atrophic scars, keloid scars, and scar contractures. Scarring is a natural and inevitable result of tissue repair. The appropriate treatment approach depends on the type and etiology of the scar [12]. Current treatments for acne scars are tailored to each patient based on the types of scars present. In cases where multiple interventions are needed, surgical sessions are typically spaced at least 4 weeks apart. Patients with acne scarring should be informed that achieving optimal results may require more than one surgical procedure [13].

Several studies have evaluated HA for treating moderate-to-severe atrophic acne scars. Twelve subjects received three sessions. Patient satisfaction was assessed over 36 weeks. The results demonstrated that HA was safe and effective, showing a gradual reduction in scar appearance, which peaked by the study's conclusion [14].

A study evaluated the safety and anti-inflammatory properties of copper tripeptide-1. The findings demonstrated its ability to strengthen the skin barrier and its effectiveness in acne prevention and treatment within cosmetic formulations. These benefits suggest that copper tripeptide-1 may play a key role in improving skin health and addressing common concerns like acne scars [15].

The clinical study investigated the effects of hesperidin on wound healing, highlighting the crucial role of fibronectin, a key extracellular matrix (ECM) component. Upon injury, fibronectin forms an initial clot with platelets and fibrin, and later aids in the development of a stable ECM, guiding tissue regeneration and remodeling throughout the healing process [16].

The clinical evaluation focused on the anti-aging effects of topically applied L-carnitine L-tartrate, a cosmeceutical active compound. The primary focus was on improving the structure of the epidermal and dermal layers of the skin. This compound offers various beneficial properties, including moisturizing effects and the enhancement of skin texture, making it highly effective in promoting skin regeneration [17].

Similarly, our study demonstrated a significant improvement in the skin scar reduction process. L-carnitine helped hydrate the skin, reducing signs of aging and improving elasticity. Copper tripeptide-1 played a key role in enhancing skin thickness, while HA maintained the skin's moisture balance. Together, copper tripeptide-1, L-carnitine L-tartrate, alpha-glucosyl hesperidin, and sodium hyaluronate provided potent antioxidant, anti-inflammatory, and skin-healing benefits. These ingredients worked synergistically to improve skin health, reduce inflammation, and enhance the overall appearance and texture of the skin. Notably, there was a reduction in skin roughness, with an increase in smoothness and a decrease in scaliness, contributing to clearer skin. The test product, Scar Fader Gel, was shown to be both safe and effective in healthy adult females, with participants reporting positive improvements in their skin’s condition. The gel effectively addressed a range of skin concerns, particularly in reducing scars and improving skin texture, promoting smoother, clearer skin overall. Importantly, no adverse events were reported during the study period, underscoring the safety of the treatment.

A key limitation of the study is its single-arm, open-label design, lacking a control, placebo, or comparator group for direct comparison, which makes it challenging to attribute observed effects such as reductions in scaliness and roughness solely to the treatment. The absence of blinding and randomization introduces potential bias, while the relatively small sample size and short duration of 45 days limit the generalizability and long-term applicability of the findings. Furthermore, environmental factors, dietary habits, and hormonal imbalances were not considered, which could have influenced the outcomes. Future research could be strengthened by incorporating a control or comparator group to minimize bias, extending the follow-up period, and including more assessment visits to enable a more comprehensive evaluation of long-term efficacy and safety. These enhancements would build upon the promising results of the current study and generate more robust, reliable, and generalizable conclusions.

The integration of Scar Fader Gel into a daily skincare routine proved highly beneficial for achieving healthier skin. Regular use of the gel contributed to significant improvements in skin texture, helping to reduce the appearance of scars while promoting smoother, more even skin. Its formulation supported the skin's natural healing process, making it an excellent addition to daily skincare regimens aimed at enhancing overall skin health. Future research should focus on assessing the long-term efficacy and safety of the treatment in larger populations. Further exploration of its effects in combination with lifestyle and environmental factors could provide additional insights into optimizing skin treatment strategies.

## Conclusions

The ThriveCo Scar Fader Gel is a safe and effective solution for managing dermal scars and improving skin texture. Enriched with Scarcede™, the formulation features a synergistic blend of active ingredients, including copper tripeptide-1, L-carnitine L-tartrate, alpha-glucosyl hesperidin, and sodium hyaluronate. These components effectively reduce scars, dryness, roughness, redness, itchiness, and scaliness, while enhancing skin smoothness and promoting a healthier, more even complexion. No adverse events or reactions were reported during the study, demonstrating the product's favorable safety profile. These findings support its efficacy and suitability for regular use in addressing skin concerns such as scars, roughness, and scaliness.

## References

[1] Monavarian M, Kader S, Moeinzadeh S, Jabbari E (2019). Regenerative scar-free skin wound healing. Tissue Eng Part B Rev.

[2] Corr DT, Hart DA (2013). Biomechanics of scar tissue and uninjured skin. Adv Wound Care (New Rochelle).

[3] Behrangi E, Goodarzi A, Rohaninasab M, Sadeghzadeh-Bazargan A, Nobari NN, Ghassemi M (2020). A review of scar treatment related to acne and burn. J Crit Rev.

[4] Connolly D, Vu HL, Mariwalla K, Saedi N (2017). Acne scarring—pathogenesis, evaluation, and treatment options. J Clin Aesthet Dermatol.

[5] Li Y, Wang SW, Liu YH, Zou MY, Wu JX, Luo SK, Hong WJ (2024). Efficacy and safety of non-cross-linked hyaluronic acid compound in the treatment of keratosis pilaris: a split-body randomized clinical trial. J Cosmet Dermatol.

[6] Necas J, Bartosikova L, Brauner P, Kolar J (2008). Hyaluronic acid (hyaluronan): a review. Vet Med.

[7] Bino A, Vicentini CB, Vertuani S (2018). Novel lipidized derivatives of the bioflavonoid hesperidin: dermatological, cosmetic and chemopreventive applications. Cosmetics.

[8] Man MQ, Yang B, Elias PM (2019). Benefits of hesperidin for cutaneous functions. Evid Based Complement Alternat Med.

[9] Foitzik K, Hoting E, Förster T, Pertile P, Paus R (2007). L-carnitine-L-tartrate promotes human hair growth in vitro. Exp Dermatol.

[10] Kalasariya HS, Pereira L, Patel NB (2023). Comprehensive phytochemical analysis and bioactivity evaluation of Padina boergesenii: unveiling its prospects as a promising cosmetic component. Mar Drugs.

[11] Dehaven Dehaven, C. C. (2014). Copper tripeptide-1. https://isclinical.com.my/wp-content/uploads/2016/06/WhitePaper_CopperTripeptide1_July2014_1_.pdf.

[12] Bayat A, McGrouther DA, Ferguson MW (2003). Skin scarring. BMJ.

[13] Jacob CI, Dover JS, Kaminer MS (2001). Acne scarring: a classification system and review of treatment options. J Am Acad Dermatol.

[14] Dierickx C, Larsson MK, Blomster S (2018). Effectiveness and safety of acne scar treatment with nonanimal stabilized hyaluronic acid gel. Dermatol Surg.

[15] Kim M, Kim J (2022 ). Assessment of stability and safety of maskne cosmetic. J Fashion Bus.

[16] Vabeiryureilai M, Lalrinzuali K, Jagetia GC (2022). NF-κB and COX-2 repression with topical application of hesperidin and naringin hydrogels augments repair and regeneration of deep dermal wounds. Burns.

[17] Fox LT (2025). PheroidTM Technology for the Topical Application of Selected Cosmeceutical Actives. https://repository.nwu.ac.za/items/e0b90779-f64a-4d4a-8aa4-acb5e8ac857b.

